# Using Public Health and Community Partnerships to Reduce Density of Alcohol Outlets

**DOI:** 10.5888/pcd10.120090

**Published:** 2013-04-11

**Authors:** David H. Jernigan, Michael Sparks, Evelyn Yang, Randy Schwartz

**Affiliations:** Author Affiliations: Michael Sparks, Sparks Initiatives, Kihei, HI; Evelyn Yang, Randy Schwartz, Community Anti-Drug Coalitions of America, Alexandria, Virginia.

## Abstract

Excessive alcohol use causes approximately 80,000 deaths in the United States each year. *The Guide to Community Preventive Services* recommends reducing the density of alcohol outlets — the number of physical locations in which alcoholic beverages are available for purchase either per area or per population — through the use of regulatory authority as an effective strategy for reducing excessive alcohol consumption and related harms.

We briefly review the research on density of alcohol outlets and public health and describe the powers localities have to influence alcohol outlet density. We summarize *Regulating Alcohol Outlet Density: An Action Guide*, which describes steps that local communities can take to reduce outlet density and the key competencies and resources of state and local health departments. These include expertise in public health surveillance and evaluation methods, identification and tracking of outcome measures, geographic information systems (GIS) mapping, community planning and development of multisector efforts, and education of community leaders and policy makers. We illustrate the potential for partnerships between public health agencies and local communities by presenting a contemporary case study from Omaha, Nebraska.

Public health agencies have a vital and necessary role to play in efforts to reduce alcohol outlet density. They are often unaware of the potential of this strategy and have strong potential partners in the thousands of community coalitions nationwide that are focused on reducing alcohol-related problems.

## Introduction

Excessive alcohol use includes binge drinking (defined as 5 or more drinks for men or 4 or more drinks for women on 1 or more occasions), heavy drinking (more than 1 drink per day on average for women or more than 2 for men), and any drinking among underage youth or women who are pregnant (1). Excessive alcohol use is the nation’s third-leading cause of preventable death, causing approximately 80,000 deaths per year in the United States (2,3) and contributing to a range of health and social problems, including automobile crashes and drowning, heart disease, hypertension, cancers such as breast and oral-pharyngeal, interpersonal violence, HIV infection, unplanned pregnancy, alcohol poisoning, and fetal alcohol spectrum disorders (4). These negative consequences for individuals, families, communities, and society at large cost the United States approximately $223.5 billion in 2006 (5).

## Regulating Alcohol Outlet Density: A Public Health Strategy

The public health profession has a tradition of promoting health and preventing harm in populations through the use of laws and regulations, including land use and zoning codes. Regulation of alcohol outlet density is part of this tradition (6). However, despite evidence supporting regulation of alcohol outlet density, many public health practitioners are unaware of its potential and do not know how to implement it.

Alcohol outlet density refers to “the number of physical locations in which alcoholic beverages are available for purchase either per area or per population” (7). Alcohol outlets include all commercial businesses that sell and serve alcohol for on-premise (eg, bars, restaurants) or off-premise consumption (eg, convenience and grocery stores).

Numerous studies have found a significant relationship between alcohol outlet density and alcohol consumption and alcohol-related harms. Examples of such findings include the following:

- In Los Angeles County, researchers estimated that every additional alcohol outlet was associated with 3.4 additional violent incidents per year (8).
- In Cleveland, researchers estimated that every additional bar added to a city block resulted in 3.4 more crimes being committed on that block per year (9).
- In New Orleans, researchers predicted that a 10% increase in the density of outlets selling alcohol for off-premise consumption would increase the homicide rate by 2.4% (10).
- Researchers in Newark, New Jersey, found an almost 1-to-1 relationship between alcohol outlets and crime; that is, a slightly less than 1% decrease in the density of alcohol outlets would result in a 1% drop in violent crime (11).

A review of 88 studies on alcohol outlet density and public health by Campbell et al (7) concluded that greater outlet density was associated with a variety of public health and safety concerns, including increased alcohol consumption, alcohol-impaired driving, injury, crime, violence, neighborhood disruption, and other harms. The review noted the relative lack of research on the health effect of reducing alcohol outlet density — most natural experiments have taken place in environments of increasing density. One study found that a decrease in the number of outlets (as a result of remonopolization, not density regulation) selling medium-strength beer in Sweden led to significant declines in hospitalizations for acute intoxication, suicides, and motor vehicle crashes (12). Studies of bans on alcohol sales in isolated communities also demonstrated the positive health effects of reducing the physical availability of alcohol (7). A nonpeer-reviewed case study of changes in land use and nuisance abatement provisions in Vallejo, California, estimated that such changes led to a 53% reduction in alcohol-outlet–related police calls for service (13).

On the basis of the evidence in the Campbell review, the independent, nonfederal Task Force on Community Preventive Services “found sufficient evidence of a positive association between outlet density and excessive alcohol consumption and related harms to recommend limiting alcohol outlet density through the use of regulatory authority (eg, licensing and zoning) as a means of reducing or controlling excessive alcohol consumption and related harms” (14).

## Using Local Zoning and Land-Use Regulations to Influence Density

States and localities can reduce alcohol outlet density in at least 4 ways:

- Limit the number of alcohol outlets per specific geographic unit.
- Limit the number of outlets per population.
- Establish a cap on the percentage of retail alcohol outlets per total retail businesses in a geographic area.
- Limit the location and operating hours of alcohol outlets.

In addition to these possibilities, localities may use land-use powers to limit, deny, or remove permission to sell alcohol from existing outlets.

Public health efforts to address problems related to alcohol outlets at the community level date back at least to 1977, when the Oakland, California, city council, recognizing a link between alcohol outlets and neighborhood crime and violence, adopted a zoning ordinance giving it the power to grant or deny land use permits for new alcohol outlets (15). Fifteen years later, backed by strong community support, the city adopted a “deemed approved ordinance,” establishing new criteria for approval of alcohol outlets under local zoning laws, approving all existing outlets automatically, and levying a fee on them that funded annual inspections to ensure that outlets were compliant with new criteria (16,17). After the ordinance won the approval of California courts (18), it set the stage for other cities across the country to exercise greater control over the operations of problematic alcohol outlets within their borders.

The City of Oakland could do what it did because the repeal of Prohibition in the United States gave states primary responsibility for decisions affecting alcohol outlet density. Many states allow local jurisdictions to impose stricter limitations through their own licensing authority or through land use (also known as zoning) and enforcement policies. State preemption is the legal doctrine that determines the degree of local control over licensing decisions that affect alcohol outlet density decisions. Local governments have authority to regulate alcohol outlet density only to the extent that the state grants that authority. States belong to various categories of preemption, which range from exclusive state preemption to exclusive local licensing, and, depending on the category, both levels of government can play important roles in regulating density. This interplay between state and local powers affects actions that states and communities decide to take (19).

In many jurisdictions, local or state licensing boards make alcohol outlet licensing decisions without input from local authorities. However, land-use decisions more typically involve local governments, because these decisions require assessment of local conditions — ensuring, for example, that the alcohol outlet location is compatible with the surrounding area and will not create a public nuisance. Public nuisance-abatement ordinances and permit processes found in local zoning ordinances (often referred to as “conditional-use permits” [CUPs]) usually govern local land use. CUPs typically regulate new alcohol outlets, whereas nuisance-abatement ordinances regulate existing outlets. Together these 2 tools can prevent overconcentration of new alcohol outlets and reduce problems with existing outlets. Examples of CUPs and nuisance- abatement ordinances are available from www.camy.org/action/outlet_density.

## An Action Guide for Reducing Alcohol Outlet Density

Following up on the recommendation of *The Guide to Community Preventive Services* (14), the Centers for Disease Control and Prevention funded the Center on Alcohol Marketing and Youth (CAMY) and the Community Anti-Drug Coalitions of America (CADCA) to develop training materials and an action guide, *Regulating Alcohol Outlet Density* (13). The 2 organizations drew on expertise in the field of alcohol policy and worked with an advisory group composed of state and local community coalition leaders and city and state public health department employees.

## Roles for State and Local Public Health Agencies and Community Coalitions

*Regulating Alcohol Outlet Density* (13) describes the unique roles state and local health departments and community coalitions can play in reducing alcohol outlet density. The more localized decision making about land use and alcohol licensing is the greater the role of state and local public health agencies to inform local decision making. State and community efforts to regulate alcohol outlet density begin with public health surveillance and measurement of the number and location of outlets, with particular attention to the distances from one to another. Surveillance can include data on binge drinking (eg, on the type of beverages consumed by binge drinkers), drinking locations, alcohol-impaired driving by adults and youth, locations where alcohol-related crimes occur, and police calls for service and the relationship of these data to specific alcohol outlets and alcohol outlet density. These data can be combined with geographic information systems (GIS) mapping to develop visual representations of the spatial connection between alcohol outlet density and community problems.

Federal funds cannot be used to lobby at the federal, state, or local level. However, federal and state prohibitions on lobbying do not prevent state and local health departments from informing policy debates. State and local health departments can provide crucial support by identifying, tracking, and providing data (eg, outcome measures) and developing GIS maps that show relationships between outlet density and community problems. They can also provide forums for community planning and conduct and sponsor education of community leaders and policy makers.

As in other areas of public health (20–22), partnerships with community coalitions are essential. Through the Drug-Free Communities program, the federal government developed a network of local coalitions skilled at mobilizing grassroots members; strengthening community collaboration; and reducing alcohol, tobacco, and other drug use (23). Coalition membership usually includes parents, staff of nonprofit organizations, city and county officials, health department staff, law enforcement officials, and health care providers.

## Nine Steps for Local and State-Level Policy Action

*Regulating Alcohol Outlet Density* describes steps community coalitions and public health departments can take to educate and inform policy makers. These steps draw from lessons learned in tobacco control and other successful public health policy initiatives (24–26). The order of steps may vary, and some steps may require more emphasis than others, depending at least in part on whether the campaign involves state- or local-level changes.

**Step 1: Assess resources needed for policy advocacy**. What is the capacity of the community undertaking the policy campaign? Although the public health department cannot take the lead on most of these steps, it can contribute to information on the community’s human resources (eg, leadership, skills), data resources, likely challenges and opposition, and technical assistance. The assessment addresses how difficult the policy change may be to achieve and how extensive the resources are for achieving it. If resources are scarce, for instance, then attempting to shut down a single alcohol outlet that research has identified as causing a public nuisance may be a more reasonable goal than passing a city ordinance.

**Step 2: Clarify the policy goal.** The key mechanism here is to develop a *policy action statement —* approximately 25 words that articulate the problem, the policy solution, what the policy will do, who will benefit from the policy, and names of policy makers who could ultimately adopt the policy.

**Step 3: Use data to inform and educate about the value of the policy.** An *issue brief* can be useful for framing the issue and the solution. A good issue brief summarizes data on the problem and the effectiveness of the proposed solution and explains the link to related community concerns (eg, underage drinking, crime). Examples of issue briefs are available from www.camy.org/action/outlet_density.

**Step 4: Seek in-kind support from an attorney with expertise in municipal or state law**. An attorney who supports the policy goal and has expertise in related local and state laws can be indispensible in drafting an ordinance, explaining preemption issues, and advising on how to advocate without violating federal or state lobbying laws. Model ordinances are available from www.camy.org/action/outlet_density.

**Step 5: Conduct media advocacy campaigns. **Outreach to the news media augments outreach to policy makers and community leaders. Media advocacy is a powerful tool for influencing the policy process (27,28).

**Step 6: Organize and mobilize grass-roots and “grass-tops” support**. This step provides a foundation for all the other steps and involves building a grass-roots base (to establish “bottom up” support and organize the voice of the community) and educating leading decision makers (to win “top-down” support) (24,29).

**Step 7: Present the evidence to support the value of enacting the proposed policy change.** The policy-making body may be elected or appointed. Public hearings often take place, and policy supporters must be ready to make their case by providing a fair and accurate summary of the costs and benefits of the proposed solution and marshaling testimony from residents, health care professionals, health department personnel, and law enforcement. Coalitions can play a vital role in mobilizing the community and preparing the presentation. Public health departments can contribute by

- Capitalizing on relationships with decision makers to educate them about the policy effects before the public hearing.
- Responding to requests for written information.
- Responding to questions from decision makers during testimony in public hearings, in the context of their role as staff.
- Providing testimony, when requested, on the health effects of the proposed policy during public hearings.
- Testifying on the benefits of the policy during public hearings when the formal position of the health department is in support.

**Step 8: Plan for implementation, enforcement, and evaluation**. The lack of a postadoption plan can ultimately undermine the entire campaign. A law is of little value if not enforced and can be difficult to sustain without evaluation of its effects. The community plays an important role in monitoring the administration of the new ordinance, which is facilitated by early planning:

- When developing the proposed policy, engage relevant agencies in discussion about effective administration and enforcement.
- Integrate implementation and enforcement steps into the policy itself.
- Identify data that can be used to evaluate the policy from health departments, law enforcement, and other organizations.
- Set up a mechanism for communication between relevant agencies and the coalition to promote cooperation and a monitoring procedure.
- Use media contacts to publicize enforcement and implementation.

**Step 9: Overcome challenges and pitfalls**. Once a policy is enacted and implementation and enforcement have begun, the community should expect challenges, including pressure to return to the status quo. Communities can anticipate and plan for such challenges by regularly monitoring the community environment, including tracking the effects of the ordinance on community health and safety and demonstrating its value. Enactment is just the beginning of implementation, monitoring, and evaluation.

## Omaha, Nebraska: a Case Study

We developed a case study of a campaign in Omaha, Nebraska, to illustrate the 9 steps in the action guide. The Omaha campaign grew out of concerns from members of the city’s Orchard Hill Neighborhood Association (OHNA) about a proposed alcohol outlet. The location of the proposed outlet was the epicenter of violent crime and nuisance behaviors in their neighborhood; more than 2,000 police calls for service within a half-mile radius of the outlet were made within 11 months. A shooting outside the outlet further catalyzed the community. When the Nebraska Liquor Control Commission (NLCC) approved the new license despite community protests, OHNA took the case to the state supreme court, which ruled in favor of the residents, ordering the NLCC to revoke the granted alcohol license and stating that the outlet should never have received a license in the first place. The court also required the NLCC to take into account environmental conditions that can make an alcohol outlet either a viable business or a factor in community disintegration (30).

This court case raised community awareness about the lack of local control over outlet density in Omaha and helped the community achieve Step 1: the community developed a sense of the resources available for policy change. The case also contributed to Step 6 (mobilization of grass-roots support). In 2010, as outlet-related crime and violence flared, Project Extra Mile (PEM), an Omaha-based nonprofit organization focused on underage drinking, helped residents to clarify their policy goal (Step 2): to pass a local land-use ordinance providing the city with the final authority to determine whether a use permit should be granted to a new alcohol outlet within city limits, thereby bypassing the NLCC process.

The campaign was dubbed “LOCAL” — “Let Omaha Control Its Alcohol Landscape” (www.thelocalcampaign.com). The county public health department provided guidance and expert testimony throughout the process. PEM used GIS maps to illustrate the problem, and residents collected personal stories of problems with the existing outlets to create testimonials (Step 3). PEM worked with a Nebraska attorney and a national legal expert on state alcohol laws (Step 4). The new Omaha ordinance included nuisance-abatement performance standards, which set the basis for complaints to the city zoning department to include actions such as disturbance of the peace, illegal drug activity, public drinking or drunkenness, harassment of passersby, public urination, assaults, vandalism, and so on. These were modeled after the provisions of Oakland’s deemed approved ordinance (Step 4). PEM parlayed its network of media contacts and media advocacy expertise into news coverage (31,32), letters to the editor, and guest opinion pieces (Step 5) (33). Meetings with city council members on the proposed ordinance (Step 7) took place throughout the process with increasing public health department support and strong grass-roots involvement. In October 2012, the Omaha City Council adopted the nuisance standards (34). Discussions about the implementation, enforcement, and evaluation of the new ordinance (Steps 8 and 9) continue. 

## Summary

Local coalitions can collaborate with state and local public health agencies to reduce excessive drinking through regulating alcohol outlet density. An action guide, *Regulating Alcohol Outlet Density*, describes 9 steps in the process (13). Public health agencies have a vital and necessary role to play in this effort, and they have strong potential partners in the thousands of community coalitions nationwide that focus on reducing alcohol-related problems. The strengths of this technique for public health action lie in the synergy that occurs when community coalitions and health departments forge partnerships. Taking advantage of this synergy, community coalitions and public health departments can use evidence-based strategies such as alcohol outlet density reduction to create healthier and safer communities.
